# Morphological Variation in Scarlet Plume (*Euphorbia fulgens* Karw ex Klotzsch, Euphorbiaceae), an Underutilized Ornamental Resource of Mexico with Global Importance

**DOI:** 10.3390/plants10102020

**Published:** 2021-09-26

**Authors:** Mónica Pérez-Nicolás, Teresa Colinas-León, Iran Alia-Tejacal, Gisela Peña-Ortega, Fernando González-Andrés, Leonardo Beltrán-Rodríguez

**Affiliations:** 1 Departamento de Fitotecnia, Universidad Autónoma Chapingo, Texcoco 56230, Mexico; mpereznicolas01@gmail.com (M.P.-N.); mgise310@gmail.com (G.P.-O.); 2 Facultad de Ciencias Agropecuarias, Universidad Autónoma del Estado de Morelos, Cuernavaca 62210, Mexico; ijac96@yahoo.com.mx; 3 Instituto de Medio Ambiente, Recursos Naturales y Biodiversidad, Universidad de León, 24009 León, Spain; fgona@unileon.es; 4 Jardín Botánico, Instituto de Biología, Universidad Nacional Autónoma de México, Ciudad de México 04510, Mexico; leonardo.beltran@ib.unam.mx

**Keywords:** cut flower, *Euphorbia*, inflorescence, Mexican endemic, morphometry, ornamental

## Abstract

Morphological variation is useful in conservation and genetic improvement programs. *Euphorbia fulgens,* a range-restricted local endemic species of Mexico, is used locally during the altars in the festivities of different saints and is also cultivated as an ornamental plant mainly in Europe. Thus, in the present study, morphological variation was evaluated in wild populations and cultivated populations. Characterization of 90 individuals from three wild populations (the only ones recorded to date) was done by measuring 30 morphological traits both vegetative and reproductive. Thereafter, seeds were collected, and established under greenhouse conditions, and 39 morphometric variables were evaluated in adult plants. An analysis of variance (ANOVA) was done for wild and cultivated groups independently, and when significant differences were found, Tukey’s comparison of means was applied (*p* < 0.05). To identify the traits responsible for the differences between wild and cultivated groups, a linear discriminant analysis (LDA) was conducted. Morphological variation was found among wild populations, and this variation decreased in cultivated populations, mainly in reproductive structures. The LDA separated the wild populations from the cultivated groups, according to inflorescence length, petiole length/blade length ratio, and leaf roundness. The variables that determined the separation of individuals between wild and cultivated populations were cyme number, foliar Feret diameter, and inflorescence length, variables that can be important for breeding strategies and artificial selection.

## 1. Introduction

Morphological variation refers to the morphometric differences observed as a result of genetic aspects and environmental factors [1,2]. Such variation has been studied in different species that have been subjected to diverse handling methods in situ and ex situ, such as agriculture, harvest, tolerance, induction, and protection [3,4,5,6].

Several authors have proposed that the domestication process of a genetic resource generates phenotypic variation, due to selection of traits that are desirable for humans, and therefore, morphological differences are evaluated between cultivated, semicultivated, and wild-growing plants [7,8,9,10].

Most of the studies in this topic, have been performed in edible plants, while a few have focused on plants used for other purposes. To date, studies in which morphological variation has been evaluated between cultivated ornamental plants and their wild relatives are scarce worldwide [11,12]. Most research has focused on characterizing different genotypes of cultivated plants or with some level of handling [13,14,15,16].

On the other hand, some researchers have studied how diverse environmental factors, such as altitude, precipitation, physicochemical characteristics of soil, light intensity, hillside orientation, among others, contribute to phenotypic variation in different species as a result of the adaptation process in distinct habitats [17,18,19,20]. Most studies have assessed wild species of broad distribution, although some have been done on microendemic species as well because due to their restricted distribution, the morphometric variation associated with edaphoclimatic characteristics can be inferred under different environmental scenarios [21,22,23,24].

*Euphobia fulgens* Karw. ex Klotzsch is a native species of Mexico, with restricted distribution and local endemic of Sierra Sur in Oaxaca state. To date, only three wild populations have been reported, located in ravines with steep slopes and of difficult access, in acidic (pH ≈ 5) loam soils. In the towns close to the places where the wild populations are found the inhabitants use the flower stems as ornaments in the altars during different saints festivities. In other countries, the common name “scarlet plume” is used to refer to several cultivated varieties of *E. fulgens*, which are produced and commercialized mainly as a cut flower and pot plant in Europe [25,26], whereas in Mexico it is not commercialized.

Due to its ornamental relevance in other countries, *E. fulgens* is a phytogenetic resource that can be exploited sustainably in Mexico, since it has esthetic values (mainly the inflorescences) and it adapts easily to cultivation conditions.

On one hand, variation assessment of wild-growing populations of *E. fulgens* is convenient, due to its local endemism, which could be the start of establishing successful conservation programs. On the other hand, the *E. fulgens* varieties are commercialized are produced abroad. Therefore, the evaluation of variation in populations of *E. fulgens* under controlled conditions could lead to the establishment of genetic improvement programs to produce tolerant varieties for the climatic conditions of Mexico, which would reduce costs, and consequently, would allow local ornamental flower producers to commercialize the plant [27]. Hence, the objective of this study was to quantify the morphological variation of *E. fulgens* in wild and cultivated populations, under the assumption that, due to their restricted distribution, the morphometric variation of wild plants will be lower in plants under cultivation conditions compared to wild-growing ones.

## 2. Results

### 2.1. Morphological Variation in Wild Populations

Significant differences were detected among wild-growing populations of *E. fulgens* (Table 1). The morphometric variables that were statistically different in the three populations evaluated were blade length and stamen length. However, when comparisons were made among pairs of populations, differences were observed in a greater number of variables. Between populations 1 and 2 there was a significant difference in 16 of the 30 morphometric variables studied. These differences correspond to 6 variables of vegetative structures and 10 of reproductive structures. Between populations 1 and 3, the variables that showed significant differences were 10 (6 of vegetative structures and 4 of reproductive structures). Finally, between populations 2 and 3, there were 12 variables that evidenced significant differences (2 in vegetative structures and 10 in reproductive structures).

**Figure 1:**
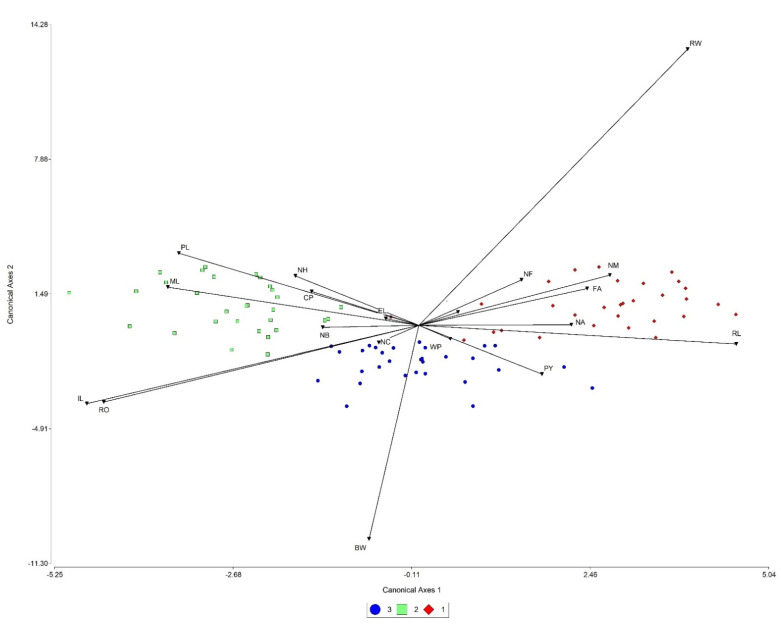
Linear discriminant analysis concerning the morphological parameters studied in the three wild populations of *Euphorbia fulgens* from Oaxaca, Mexico.

The graphical representation of the LDA showed that canonical axis 1 explained 73.76% of the variation between groups and canonical axis 2 the other 26.24 % (Figure 1). Both axes were significant (*p* < 0.001), demonstrating a clear differentiation among populations. The most opposite populations were 1 and 2. From the first standardized discriminant function by common covariances, it can be observed that inflorescence length (−0.87) is the most relevant variable for discrimination over this axis, followed by the petiole length/blade length ratio (0.83) and leaf roundness (−0.83). In axis 2, the most relevant variables for discrimination among groups were the blade width/blade length ratio (2.39), blade width (−1.85), and inflorescence length (−0.68). The apparent mean error rate in the classification of groups was low (4%).

### 2.2. Morphological Variation in Cultivated Populations

Lower morphological variation was detected in cultivated populations of *E. fulgens* (Table 2) compared to wild populations (Table 1). The significantly different variables between the three cultivated populations were blade width and the blade width/blade length ratio; whereas between populations 1 and 2 were nine (six of vegetative structures and three of reproductive structures). Compared to the wild populations, variation in vegetative structures of the cultivated populations was similar, whereas in reproductive structures the variation was lower and only found in the number of female cyathia and blade colour of leaves in the upper third.

Meanwhile, nine morphometric variables (six of vegetative structures and three of reproductive structures) were significantly different between populations 1 and 3. Most variables of vegetative structures showed a variation similar to that recorded in the wild populations, while only one variable of reproductive structures (stamen length) had a similar variation.

Finally, the significantly different variables between populations 2 and 3 were seven, where the longest cyme length and basal diameter were also significantly different in wild-growing populations. In summary, the variation found in vegetative structures of wild-growing and cultivated populations was similar, while the variation in reproductive structures decreased considerably in cultivated populations.

### 2.3. Morphometric Variation between Wild-Growing and Cultivated Populations of E. fulgens

**Figure 2:**
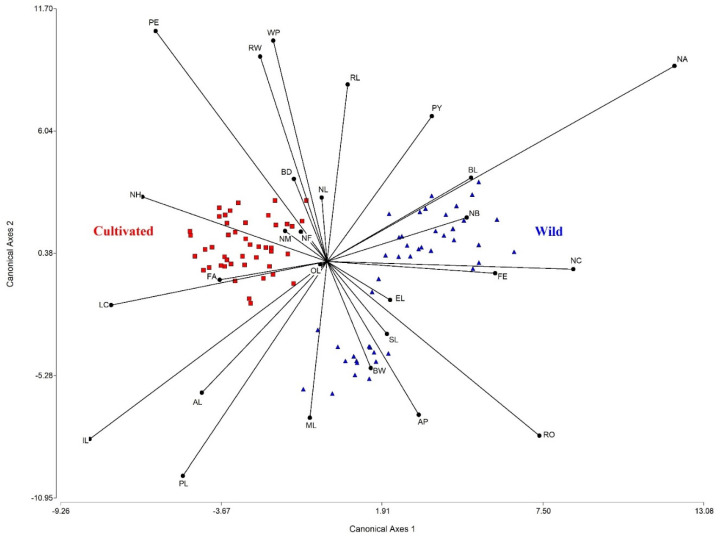
Linear discriminant analysis of morphometric variables between three wild-growing and cultivated populations of *Euphorbia fulgens* of Oaxaca, Mexico.

LDA indicated a clear differentiation between wild-growing and cultivated populations of *E. fulgens* (Figure 2). Both canonical axes were significant (*p* < 0.001), where axis 1 explained 48.65% of the variation between groups, whereas axis 2 explained 24.44%. It could be observed that the number of cymes (1.48) was the most relevant variable for discrimination over axis 1, followed by Feret diameter (−1.05) and inflorescence length (−1.01). In axis 2, the most relevant variables for discrimination were leaf perimeter (1.31), petal appendage width (1.25), and petiole length (−1.22).

## 3. Discussion

### 3.1. Morphological Variation in Wild Populations

Differences between wild-growing populations were observed in most vegetative and reproductive structures, even though *E. fulgens* has a restricted distribution with only three populations reported so far. Populations 1 and 2 were the most contrasting in leaf shape and size, as well as in inflorescence size and colour. Among the three populations, populations 1 and 2 were the most distant (80 km apart) and belonged to similar vegetation types but in different altitudes and on areas with different edaphoclimatic characteristics (Table 1). Moreover, populations 2 and 3 contrasted mostly in reproductive structures, but shared edaphoclimatic characteristics and were located at a similar altitude. However, the three populations did not show outstanding differences. This suggests that phenotypic variation is probably determined by edaphoclimatic factors.

The obtained results agree with those found by other authors. It has been reported that altitude influenced the leaf morphology of *Yucca capensis*, an endemic species of Baja California Sur, Mexico [22], as well as in cone and needle morphology of pines [21]. Likewise, the altitude had an effect on leaf and fruit morphology of shea tree [28]. Furthermore, in a study of soil physicochemical properties, differences were observed in height and leaf size of Iroko trees [18].

Other factors that were not analyzed in this study but have been observed to have an impact on morphometric variation of vegetative and reproductive structures, were ground slope, soil depth, stony soil, temperature, wind speed, rain, and relative humidity [29,30]. In contrast, in other studies conducted in members of the genus *Opuntia*, it was determined that phenotypic differences in cladodes between populations were more related to population density than environmental factors [31]. Therefore, in future studies, it is suggested to increase the number of variables to determine their effects and, consequently, obtain more information for the design of propagation or agronomic management experiments.

The information gathered in this study about the morphological variation between wild-growing populations of *E. fulgens* will contribute to the establishment of in situ conservation programs of a germplasm that is not found in any other part of the world. Endemic species are susceptible to climate change because their restricted distribution is associated with specific environmental conditions. Hence, more studies should be conducted to decipher the adaptations of the local endemic plants in different predicted environmental scenarios in order to establish concrete management plans, as in other endemic species [23,24].

### 3.2. Morphological Variation in Cultivated Populations

The cultivated populations of *E. fulgens* in greenhouse showed a morphometric variation in vegetative structures, similar to the wild-growing groups, which suggests that these characteristics are highly stable in this species. In most studies of ornamental plants, phenotypic differences in structures with aesthetic or commercial value are evaluated; for instance, stem length, firmness, and flower number, size, colour, and odor in Peruvian lily [15]; branch number and length, flowering duration, height, disc and ligulate flowers, and central head diameter in sunflower [14]; and growth habit, ramification, foliar blade, and fruit shape, size, and colour in ornamental gourds to obtain ideal genotypes for cut flower, bouquets, arrangements, or pots [16]. Cultivation of *E. fulgens* in greenhouse demonstrated that it adapts and develops well under controlled conditions. Thus, a genetic improvement program could be initiated focused on using this plant in a sustainable way since it has only been exploited in other countries. In this first cultivation cycle, individuals with desirable traits were identified; for instance, some with longer panicles, greater cyathium diameters, higher number of cymes, and attractive size and colour of petal appendages that could be used as progenitors of interesting features in breeding strategies. Thus, it is suggested to evaluate the effect of agronomic management variables for the probable cultivation of *E. fulgens* in the greenhouse.

### 3.3. Morphological Variation between Wild-Growing and Cultivated Populations

The differences between individuals of wild-growing and cultivated populations of *E. fulgens* were determined by number of cymes, petal appendage width, inflorescence length, petiole length, leaf perimeter, and leaf Feret diameter. Cultivated plants presented larger inflorescences and higher number of cymes, which could be the result of applying fertilizer. In contrast, the variables that determined the differences between wild-growing and cultivated individuals of Peruvian lily were flower colour and stem length [11].

In this study, we observed that, from a first cultivation cycle without an artificial selection process, there were morphological differences between wild-growing and cultivated plants of *E. fulgens*. Several authors consider that the morphometric differences and group separation of wild-growing and cultivated individuals is due to the domestication process, which was the case in *Chrysophyllum cainito* L. (star apple), where the differences in fruit and seed characteristics were caused mainly by the artificial selection process of fruits [32]. This was also observed in *Crescentia cujete* L., where due to its use as bowls, a strong selection of larger fruits, rounder forms, and shorter and wider peduncles has been done [9,33]. Differences have also been found between wild-growing and cultivated poinsettia in leaf size, internode distance, stem length and diameter; however, the leaf area/stem volume ratio has remained constant, despite the domestication process [12]. Evaluation of variation in wild-growing populations of a species that is intended to be introduced in cultivation in Mexico, will allow to perform a follow-up of how morphology is modified with handling and selecting desirable traits.

## 4. Materials and Methods

### 4.1. Morphological Variation in Wild-Growing Populations

**Figure 3:**
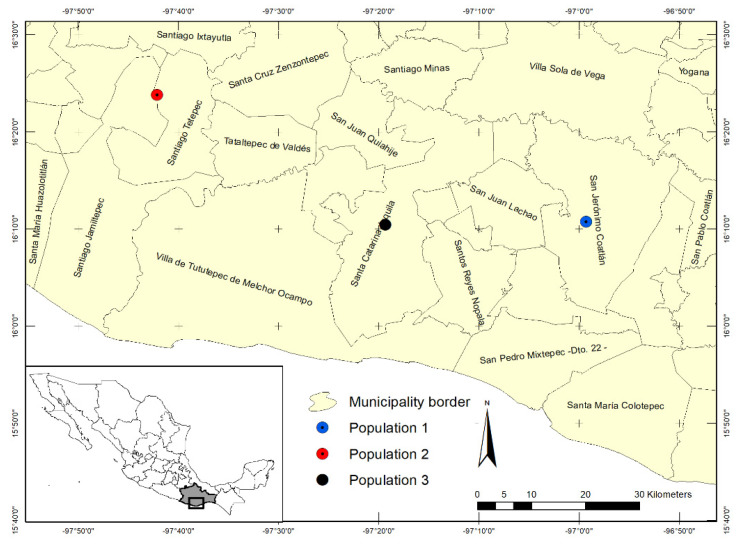
Geographical location of wild-growing populations of *Euphorbia fulgens* in Mexico.

The study was carried out in three wild-growing populations of *E. fulgens*, located in the municipalities of San Jerónimo Coatlán, Santa Catarina Juquila, and in the limits between Santiago Jamiltepec and Santiago Tetepec of Oaxaca state, Mexico, and it was based on examination of herbarium specimens and two local specialists in Euphorbiaceae. A distribution map of these populations is illustrated in Figure 3. Altitude and soil characteristics of each municipality are shown in Table 3.

**Figure 4:**
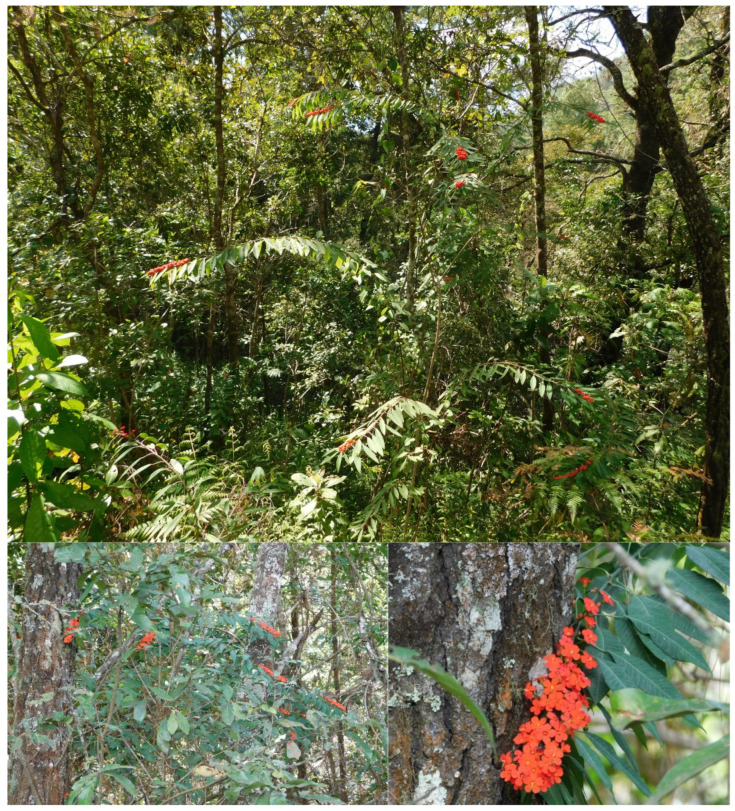
Wild-growing populations of *Euphorbia fulgens* from Oaxaca, Mexico.

Between December 2017 and January 2018, the wild-growing populations were visited (Figure 4). Transects were made in a horizontal line each 50 m. The closest individual was selected every 10 m and 30 individuals of each population were sampled in total, in an area of one hectare. The morphometric variables registered for each individual were 11 vegetative structures and 19 reproductive structures. First, the plant’s height—AL (cm) and basal diameter—BD (cm) were measured, and the number of branches—NB was counted. Thereafter, three leaves from the middle third of the stem were cut and placed on a white paper to measure petiole length—PL (cm), blade length—BL (cm), and blade width—BW (cm), as well as to take pictures with a Nikon Coolpix B500 camera. With the resulting images, the foliar area—FA (cm^2^), perimeter—PE (cm), Feret diameter—FE, and roundness—RO were calculated by using the program Image J v. 1.8.0_112 [34]. In addition, the ratios blade width/blade length—RW and petiole length/blade length—RL were calculated.

**Figure 5:**
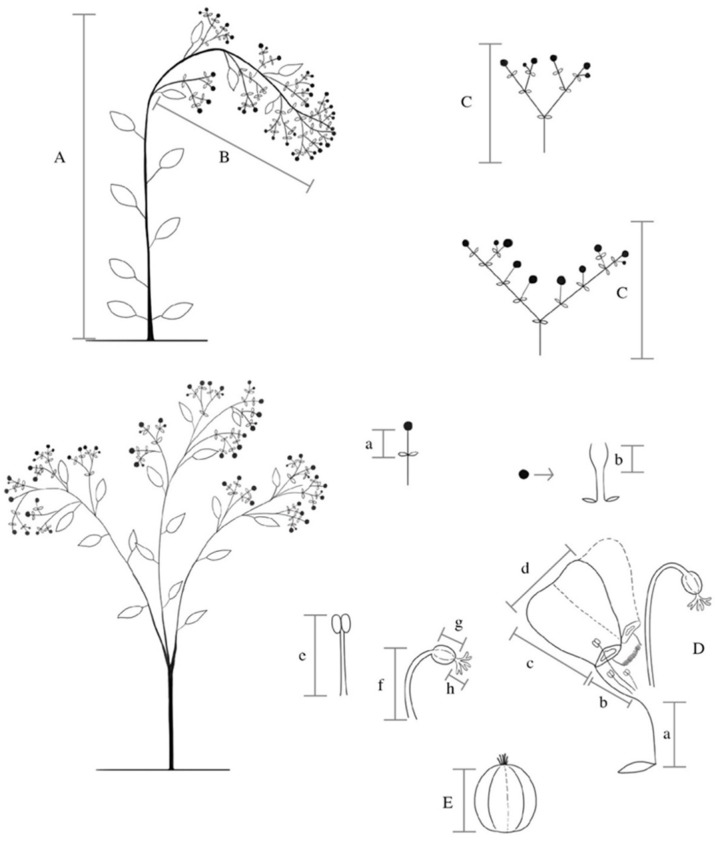
Morphometric variables evaluated in *Euphorbia fulgens*. (A) Plant height. (B) Inflorescence length or synflorescence (level 3). (C) Cyme length (level 2). (D) Cyathium (level 1). (a) peduncle length, (b) involucre length, (c) petal appendage length, (d) petal appendage width, (e) stamen length, (f) pedicel length, (g) ovary length, (h) style length, (i) gland. (E) Fruit length.

Inflorescences were organized in three levels. The first level corresponds to the cyathium, the second to the cymes, and third to the inflorescence, i.e., the whole set of inflorescences (Figure 5) [35]. In this study, inflorescence refers to the latter level of organization. Inflorescence length—IL (cm), number of cyathia per axillary cyme—NC, longest cyme length—LC (cm), number of axillary cymes—NB, number of hermaphroditic cyathia—NH, and number of male—NM and female cyathia—NF were registered. Three cyathia were cut and we measured their peduncle length—PY (cm), involucre length—NL (cm), length and width of petal appendage—AP, WP (cm), pedicel length—EL (cm), ovary length—OL (cm), style length—SL (cm), and stamen length—ML (cm). Based on the suggestion of the technical guide for *E. fulgens* varieties of the International Union for the Protection of New Varieties of Plants [36], the colour of petal appendages—CP, blade colour of leaves at the superior and inferior thirds of the inflorescence (green or reddish)—SC, IC, and red colour intensity—RC when present (light, medium, or dark) were recorded by using the Royal Horticultural Society colour charts [37].

### 4.2. Morphological Variation of Cultivated Populations of E. fulgens

In April 2018, fruits from the same wild-growing populations were collected. From each plant, according to the total number of fruits only 30% of the fruits were collected to avoid population alteration. The fruits were placed in paper bags, where they were left to dry at room temperature until the seeds were released by dehiscence. Dissection forceps were used to obtain the seeds from fruits that did not open naturally. The seeds were planted in a crystal greenhouse located in the Institute of Horticulture at the Universidad Autónoma Chapingo, Texcoco, Estado de Mexico, Mexico, at 19°29′ N, −98°53′ W, and an altitude of 2450 m. The number of seeds collected varied among populations: 100 seeds from populations 1 and 2, and 60 seeds from population 3. Seeding was done on May 10 2018 in pots (7.5 cm × 5.5 cm × 5.1 cm) filled with a peat moss/perlite substrate (50:50 *v*/*v*). Thirty-five seedlings were obtained from population 1, 67 from population 2, and 15 from population 3. Seedlings were irrigated with the starter fertilizer Ultrasol^®^ N15-P30-K15+M.E. AQTEX.

When the fourth true leaf emerged (in June), seedlings were transplanted to black polyethylene bags (25 cm × 25 cm), filled with a substrate of black soil/oak leaf soil/pine bark/coconut fiber/vermicompost (20:30:20:20:10 *v*/*v*), and were placed under a polyethylene shade cloth at 50%. Multipurpose fertilizer Ultrasol^®^ N18-P18-K18 + M.E. AQTEX was added to the irrigation water. To prevent the growth of fungi, benomyl was applied (Promyl^®^, 1.5 g L^−1^), and to prevent black fly, bifenthrin was used (Talstar^®^, 1.5 ml L^−1^). Weed control was done manually.

**Figure 6:**
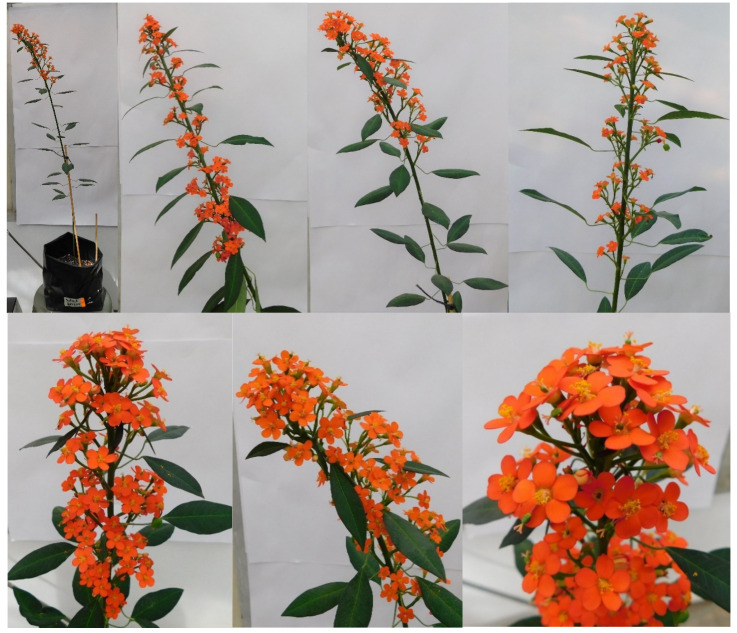

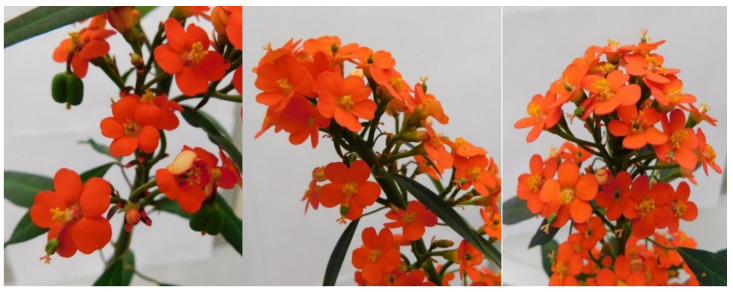
Various aspects of the inflorescences of *Euphorbia fulgens* during its first cultivation cycle under greenhouse conditions in Texcoco, Mexico.

In January, data were gathered when the plants had one or two fruits. Fifteen plants per population were chosen randomly, since this was the highest number of plants obtained from population 3 (Figure 6). The same 14 vegetative structures and 25 reproductive variables evaluated in wild-growing populations were recorded, and due to the ease of working in greenhouse, 9 variables were added: internode length—IE (cm), number of leaves prior to inflorescence—NA, internode length in inflorescences—NI (cm), inflorescence diameter (cyathium)—DI (cm), fruit length—FL (cm), fruit diameter—FD (cm), fruit number—FN, number of seeds—NS, and number of sprouts after harvesting the fruits—NZ.

### 4.3. Statistical Analysis

To determine significant differences between *E. fulgens* populations, analysis of variance (ANOVA) was done for each of the 30 morphometric variables measured in the field and the 39 variables quantified in greenhouse, followed by comparison of means with Tukey’s test (*p* < 0.05). Before statistical analysis was applied, all variables were standardized by dividing the mean by the standard deviation. Normality (Shapiro-Wilks) and homoscedasticity tests (Kolmogorov) were applied, and all variables met these assumptions, so the parametric analysis was carried out.

To identify the variables responsible for the spatial separation in the wild-growing and cultivated groups defined a priori, a linear discriminant analysis (LDA) was done [38], where the dependent variable was the population under study and the independent variables were the morphometric parameters. Previously, a principal component analysis (PCA) was applied to wild-growing and cultivated populations separately to eliminate highly correlated variables and with a lower factor loading [38]. Consequently, from the initial 28 morphological variables, only 19 were analyzed with LDA: number of branches with flowers, petiole length, blade length, blade width/blade length ratio, petiole length/blade length ratio, foliar area, leaf roundness, inflorescence length, number of cyathia per cyme, number of axillary cymes, number of hermaphrodite cyathia, number of male cyathia, number of female cyathia, peduncle length, petal appendage width, pedicel length, style length, stamen length, and appendage colour. All analyses were done with the statistical software InfoStat, version 2020 [39].

## 5. Conclusions

*E. fulgens* is a local endemic species of Oaxaca, Mexico with restricted distribution that presents morphological variation in vegetative structures (leaves) and reproductive structures (inflorescences), this is due to different altitudes and on areas with different edaphoclimatic characteristics. Morphological variation decreased under greenhouse conditions, mainly in reproductive structures, although it was the first growing cycle with no breeding. The information in this study is useful to establish in situ conservation programs of a germplasm that is not found wild-growing in other regions, as well as to start genetic breeding in Mexico, focused on its aesthetic values and easy adaptation to cultivation.

## Figures and Tables

**Table 1:** Morphometric variables of three wild populations of *Euphorbia fulgens* from Oaxaca, Mexico.

Variable	*p*-Value	Population 1	Population 2	Population 3
Plant height	0.0696	145.53 ^a^	158.63 ^a^	180.63 ^a^
Basal diameter	0.0240	0.78 ^ab^	0.64 ^b^	1.02 ^a^
Petiole length	0.0007	3.46 ^a^	2.94 ^b^	2.95 ^b^
Blade length	0.0001	7.99 ^c^	9.03 ^b^	9.73 ^a^
Blade width	0.0204	2.75 ^a^	2.36 ^b^	2.45 ^ab^
Blade width/Blade length	0.0001	0.35 ^a^	0.26 ^b^	0.25 ^b^
Petiole length/Blade length	0.0001	0.43 ^a^	0.33 ^b^	0.30 ^b^
Leaf area	0.8267	14.43 ^a^	13.77 ^a^	14.08 ^a^
Leaf perimeter	0.4161	27.88 ^a^	27.46 ^a^	28.73 ^a^
Leaf Feret diameter	0.0504	11.12 ^b^	11.58 ^ab^	12.01 ^a^
Leaf roundness	<0.0001	0.34 ^a^	0.26 ^b^	0.25 ^b^
Number of branches with inflorescences	0.0037	4.20 ^a^	1.80 ^b^	4.07 ^a^
Inflorescence length	0.0080	12.07 ^b^	22.92 ^a^	18.72 ^ab^
Number of cyathia per axillary cyme	0.1527	3.03 ^a^	3.37 ^a^	3.07 ^a^
Longest cyme length	0.0068	2.72 ^ab^	2.87 ^a^	2.41 ^b^
Number of axillary cymes	0.9085	14.27 ^a^	13.40 ^a^	13.73 ^a^
Number of hermaphrodite cyathia	0.0001	1.53 ^b^	9.30 ^a^	0.73 ^b^
Number of male cyathia	0.0011	14.33 ^a^	7.13 ^b^	8.17 ^b^
Number of female cyathia	0.5249	1.03 ^a^	0.60 ^a^	0.67 ^a^
Peduncle length	0.0001	1.53 ^a^	1.05 ^b^	1.51 ^a^
Involucre length	0.0007	0.31 ^b^	0.30 ^b^	0.33 ^a^
Petal appendage length	0.6668	0.54 ^a^	0.54 ^a^	0.53 ^a^
Petal appendage width	0.0044	0.50 ^a^	0.50 ^a^	0.44 ^b^
Pedicel length	0.0033	0.98 ^a^	0.85 ^b^	1.02 ^a^
Ovary length	0.0136	0.32 ^a^	0.29 ^b^	0.31 ^ab^
Style length	0.0117	0.15 ^a^	0.12 ^ab^	0.11 ^b^
Stamen length	<0.0001	0.46 ^c^	0.67 ^a^	0.53 ^b^
Petal appendages colour	<0.0001	1.57 ^b^	3.20 ^a^	1.30 ^b^
Leaf blade colour in the superior third of inflorescence	0.3721	1.00 ^a^	1.00 ^a^	1.03 ^a^
Intensity of leaf blade colour in the superior third of inflorescence	0.3721	1.00 ^a^	1.00 ^a^	1.13 ^a^

Different letters in the same row indicate statistically significant difference by Tukey’s test (*p* < 0.05).

**Table 2:** Morphometric variables of cultivated *Euphorbia fulgens* plants in greenhouse conditions (Texcoco, Mexico), originating from three wild-growing populations of Oaxaca, Mexico.

Variable	*p*-Value	Population 1	Population 2	Population 3
Plant height	0.0007	88.50 ^b^	83.69 ^b^	110.87 ^a^
Basal diameter	0.0102	0.44 ^b^	0.44 ^b^	0.49 ^a^
Petiole length	0.0024	3.25 ^a^	2.73 ^b^	2.60 ^b^
Blade length	0.0032	7.74 ^b^	8.51 ^ab^	8.88 ^a^
Blade width	<0.0001	2.41 ^a^	1.38 ^c^	1.92 ^b^
Blade width/Blade length	<0.0001	0.32 ^a^	0.15 ^c^	0.21 ^b^
Petiole length/Blade length	<0.0001	0.43 ^a^	0.33 ^b^	0.29 ^b^
Internode length	0.0712	1.77 ^a^	1.95 ^a^	2.08 ^a^
Leaf area	0.0002	13.58 ^a^	9.38 ^b^	12.59 ^a^
Leaf perimeter	0.7648	29.44 ^a^	29.50 ^a^	30.11 ^a^
Leaf Feret diameter	0.0422	10.61 ^b^	11.20 ^ab^	11.55 ^a^
Leaf roundness	<0.0001	0.31 ^a^	0.17 ^b^	0.21 ^b^
Number of branches with inflorescences	0.0877	31.13 ^a^	25.87 ^a^	28.47 ^a^
Inflorescence length	0.1890	20.13 ^a^	16.97 ^a^	21.92 ^a^
Number of cyathia per axillary cyme	0.0180	3.47 ^ab^	2.93 ^b^	3.93 ^a^
Longest cyme length	0.0471	4.23 ^ab^	4.74 ^a^	3.77 ^b^
Number of axillary cymes	0.1277	14.33 ^a^	10.67 ^a^	12.40 ^a^
Number of hermaphrodite cyathia	0.0633	33.87 ^a^	20.60 ^a^	29.80 ^a^
Number of male cyathia	0.5016	14.47 ^a^	11.13 ^a^	10.40 ^a^
Number of female cyathia	0.0298	5.87 ^a^	2.13 ^b^	4.87 ^ab^
Internode length in inflorescence	0.2370	1.05 ^a^	1.12 ^a^	1.87 ^a^
Peduncle length	0.1880	1.13 ^a^	1.39 ^a^	1.19 ^a^
Involucre length	0.8454	0.32 ^a^	0.32 ^a^	0.33 ^a^
Cyathium diameter	0.2550	1.40 ^a^	1.41 ^a^	1.27 ^a^
Petal appendage length	0.0352	0.55 ^a^	0.54 ^a^	0.45 ^a^
Petal appendage width	0.7939	0.49 ^a^	0.51 ^a^	0.48 ^a^
Pedicel length	0.0636	0.83 ^a^	0.89 ^a^	0.73 ^a^
Ovary length	0.2002	0.30 ^a^	0.30 ^a^	0.27 ^a^
Style length	0.3040	0.10 ^a^	0.12 ^a^	0.11 ^a^
Stamen length	0.0156	0.70 ^a^	0.68 ^ab^	0.59 ^b^
Petal appendages colour	0.5167	3.40 ^a^	3.53 ^a^	3.07 ^a^
Leaf blade colour in the superior third of inflorescence	0.0018	1.60 ^a^	1.20 ^b^	1.00 ^b^
Intensity of leaf blade colour in the superior third of inflorescence	0.0011	2.87 ^a^	1.53 ^b^	1.07 ^b^
Fruit length	0.1489	0.69 ^a^	0.67 ^a^	0.60 ^a^
Fruit diameter	0.5954	0.60 ^a^	0.58 ^a^	0.56 ^a^
Fruit number	0.6511	5.67 ^a^	4.40 ^a^	4.93 ^a^
Seed number	0.9118	11.00 ^a^	10.40 ^a^	9.80 ^a^
Number of vegetative sprouts	0.2209	6.07 ^a^	4.47 ^a^	4.40 ^a^

Different letters in the same row indicate statistically significant difference by Tukey’s test (*p* < 0.05).

**Table 3:** Main physical characteristics of the studied wild-growing populations of *Euphorbia fulgens* sampled in Oaxaca, Mexico.

Population	Altitude (msnm)	Vegetation Type	Sand	Silt	Clay	Organic Matter
1	1421	OPF	53.3	29.3	17.2	4.48
2	1130	OPF	55.5	31.3	13.2	7.07
3	1160	MMF, SVMMF	65.9	16.0	18.1	7.54

Abbreviations: OPF = pine-oak forest, MMF = cloud forest, SVMMF = secondary vegetation of cloud forest.

## Data Availability

The datasets generated during and/or analyzed during the current study are available from the corresponding author on reasonable request.
